# Supplementary material for: Cocktail Effect of Endocrine Disrupting Chemicals: Application to Chlorpyrifos in Lavender Essential Oils
Source: Int J Environ Res Public Health. 2022 Oct 10;19(19):12984. doi: 10.3390/ijerph191912984 (PMC9566273; doi:10.3390/ijerph191912984)
Supplement: Supplementary file 1 [file ijerph-19-12984-s001.zip › IJERPH 20220907 supplemental data.pdf]

Article

Cocktail effect of endocrine disrupting-chemicals: application to chlorpyrifos in lavender essential oils

Sophie Fouyet [1,2,*], Elodie Olivier [1], Pascale Leproux [1], Sabrina Boutefnouchet[1], Mélody Dutot [1,3] and Patrice Rat [1]

[1] Université de Paris, CNRS CiTCoM, 75006 Paris, France;
[2] Laboratoires Léa Nature, 17180 Périgny, France
[3] Yslab, Recherche & Développement, 29000 Quimper, France
* Correspondence: sophie.fouyet@etu.u-paris.fr

Supplementary data

1. EOs composition analysis

1.1. General

GC-MS analysis were performed using GCMS-QP2010 Ultra (Shimadzu Co. Kyoto. Japan) equipped with an Agilent 5973N MS detector. GC-FID analysis was performed using GC-FID Agilent Technologies 7820A equipped with a Parker-Balston hydrogen generator. Analysis was developed using DB-5 (60m x 0.25mm x 0.25µm) and DB-WAX (60m x 0.25mm x 0.25µm) columns for GC-MS analysis and DB-WAX (60m x 0.25mm x 0.25µm) for GC-FID analysis.

1% solutions of Lavender 1 and Lavender 2 EOs in ethyl acetate were prepared and injected for analysis. The following analytical conditions were used for both apparatus and columns: oven temperature was programmed at 50°C, hold for 5 min, then increased to 240°C at a rate of 3°C/min, injector temperature was set at 240°C; carrier gas was helium with a flow rate of 1mL/min; splitting ratio of 1:50 was applied; injection volume was set to 1µL.

For GC-MS mass spectrometry interface, the MS source temperature was set at 220°C with an ionization energy of 70eV and interface temperature of 240°C. Full scan was recorded (50-700m/z).

Identification of constituents was achieved by comparing GC-MS mass spectra with the Mass Spectra Library (NIST 98) components and by comparison of retention indices (RI) calculated from injection of C8-C20 hydrocarbons alkanes mixture with retention indices from the literature on DB-5 column using the following formula: *RI=(100*n)+200*((RTi-RTn-1)/(RTn-RTn-1))*

Where retention indices RI are calculated using retention time of each compound (RTi), and retention time of alkanes from C8 to C20, preceding (RTn-1) and following (RTn) the considered peak.

Some selected pure standards were also used to confirm the identification of main compounds.

% of main constituents were calculated on chromatograms obtained by GC-FID analysis on the DB-WAX column (60 m x 0.25 mm x 0.25 µm).

1.2. GC-MS Chromatograms

1.2.1. GC-MS Chromatograms – DB-5 column

1.2.1.1. Lavender 1

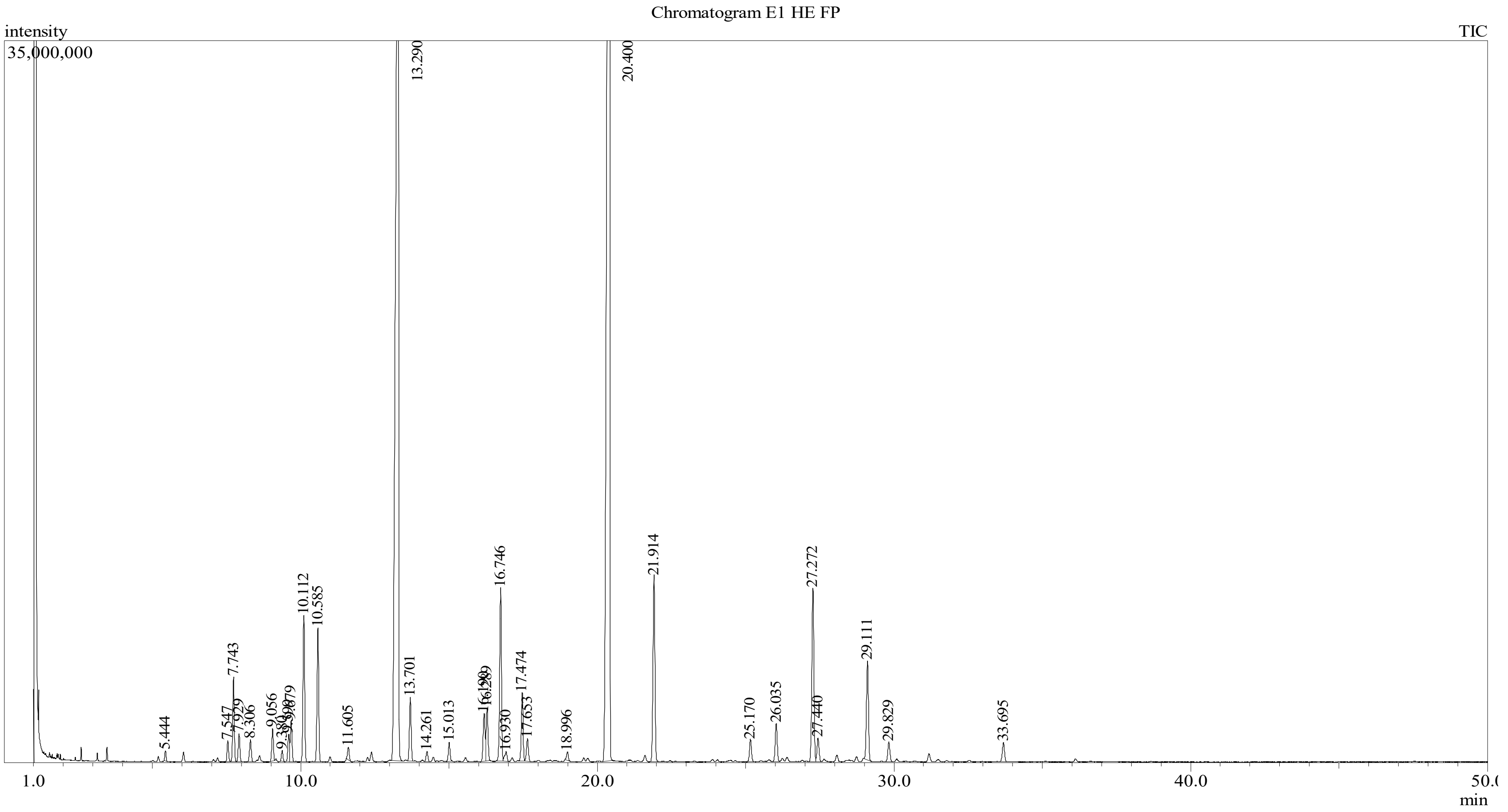

1.2.1.2. Lavender 2

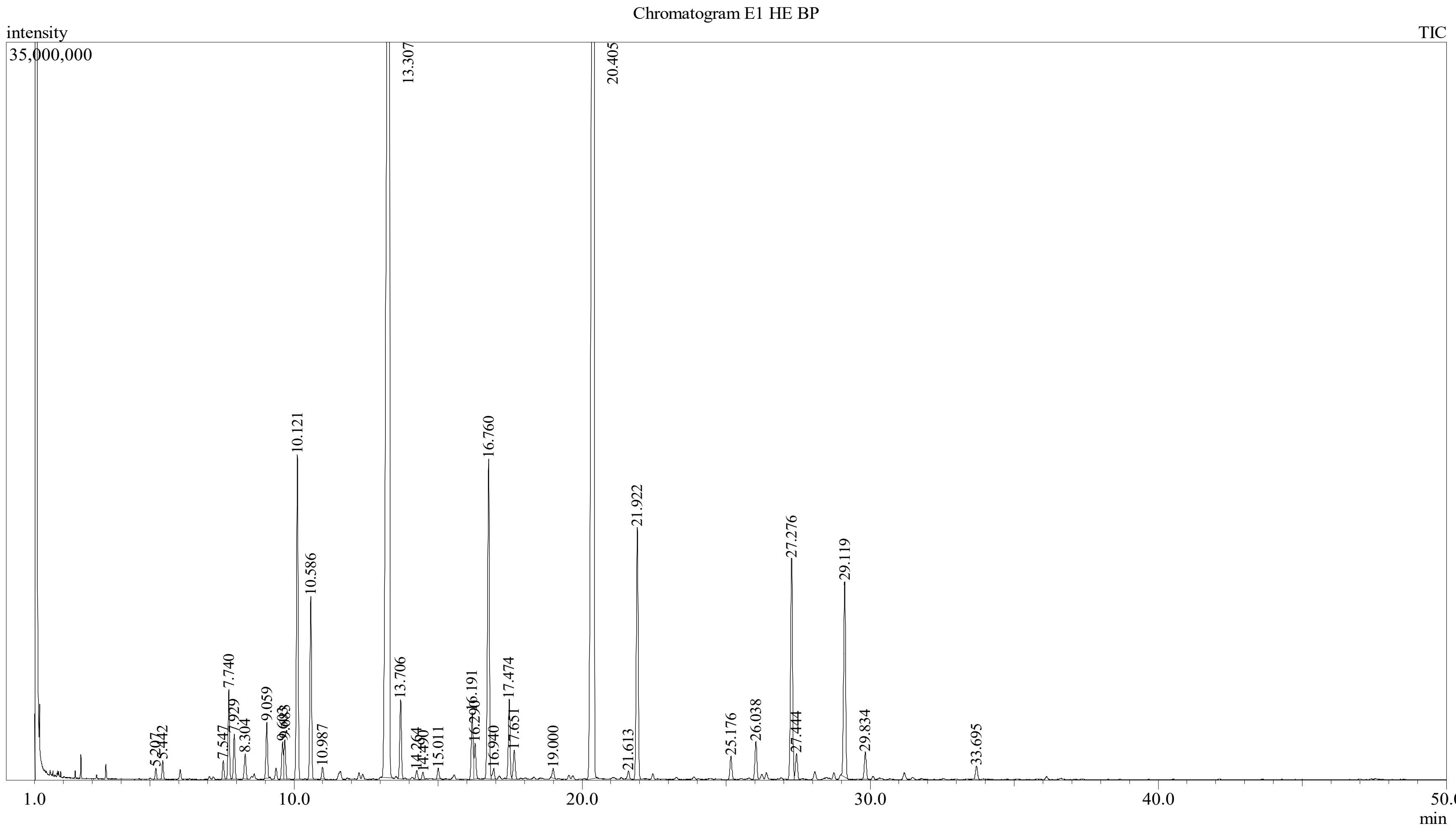

1.2.2. GC-MS Chromatograms – DB-Wax column

1.2.2.1. Lavender 1

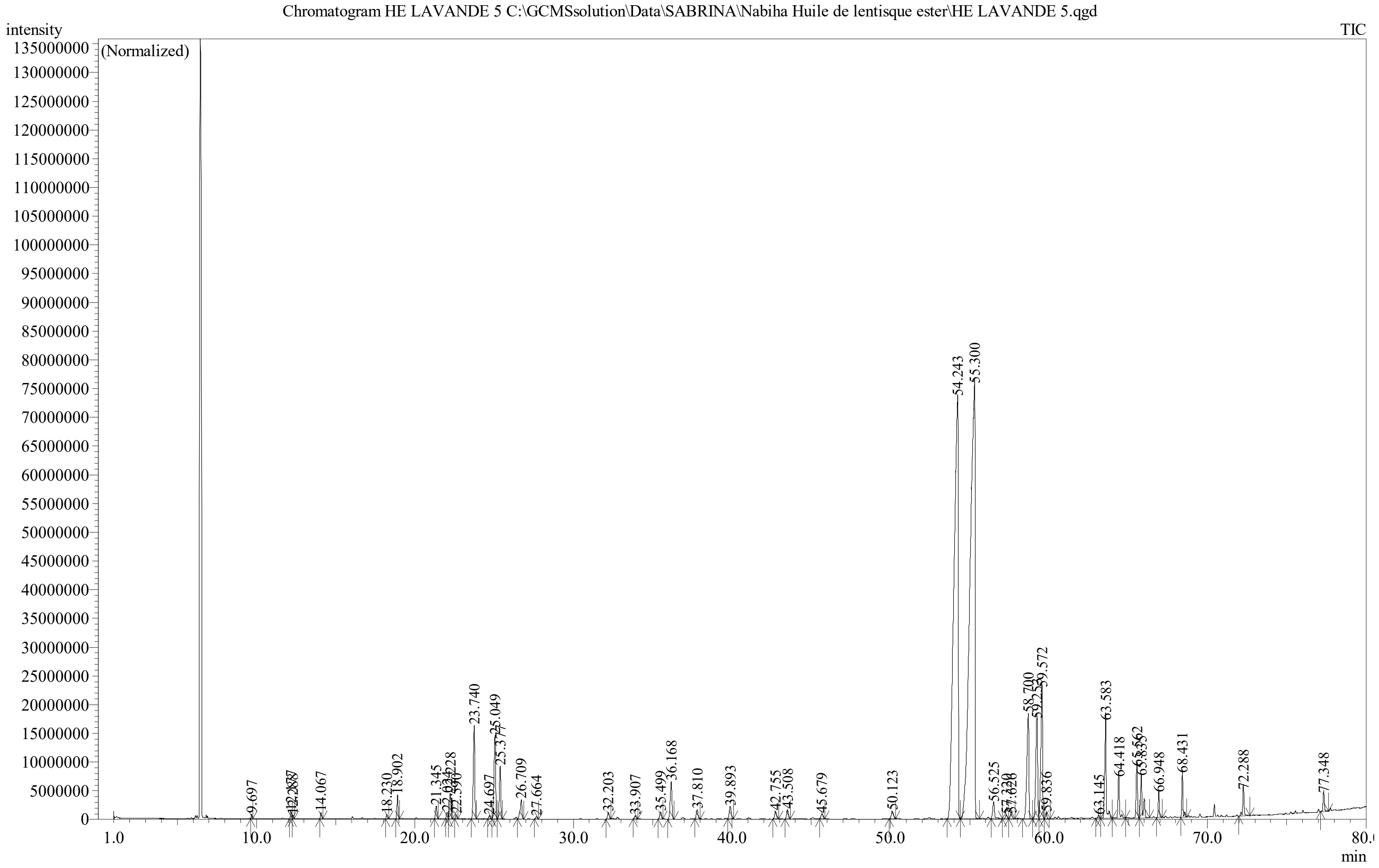

1.2.2.2. Lavender 2

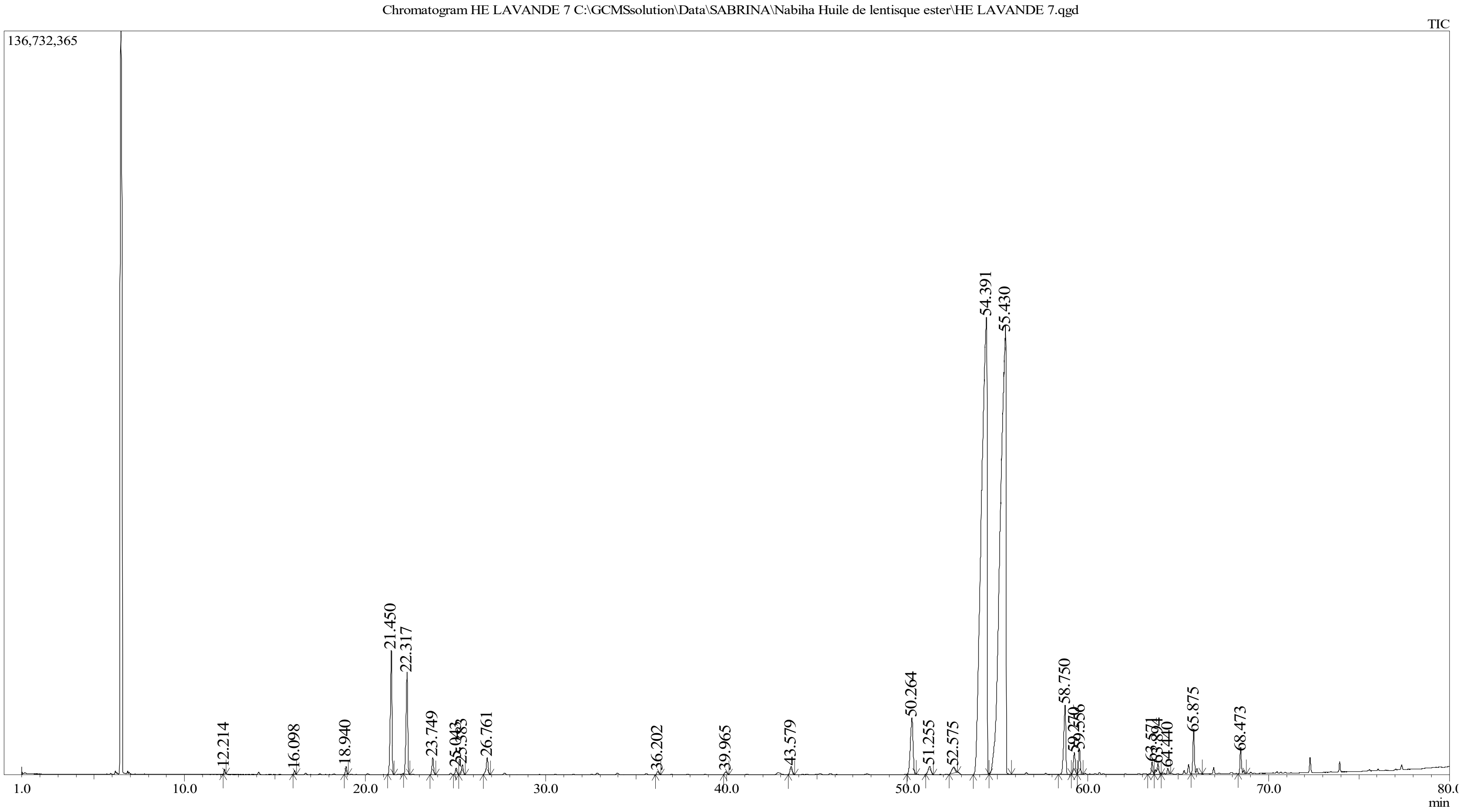

1.2.3. GC-FID Chromatograms – DB-Wax column

1.2.3.1. Lavender 1

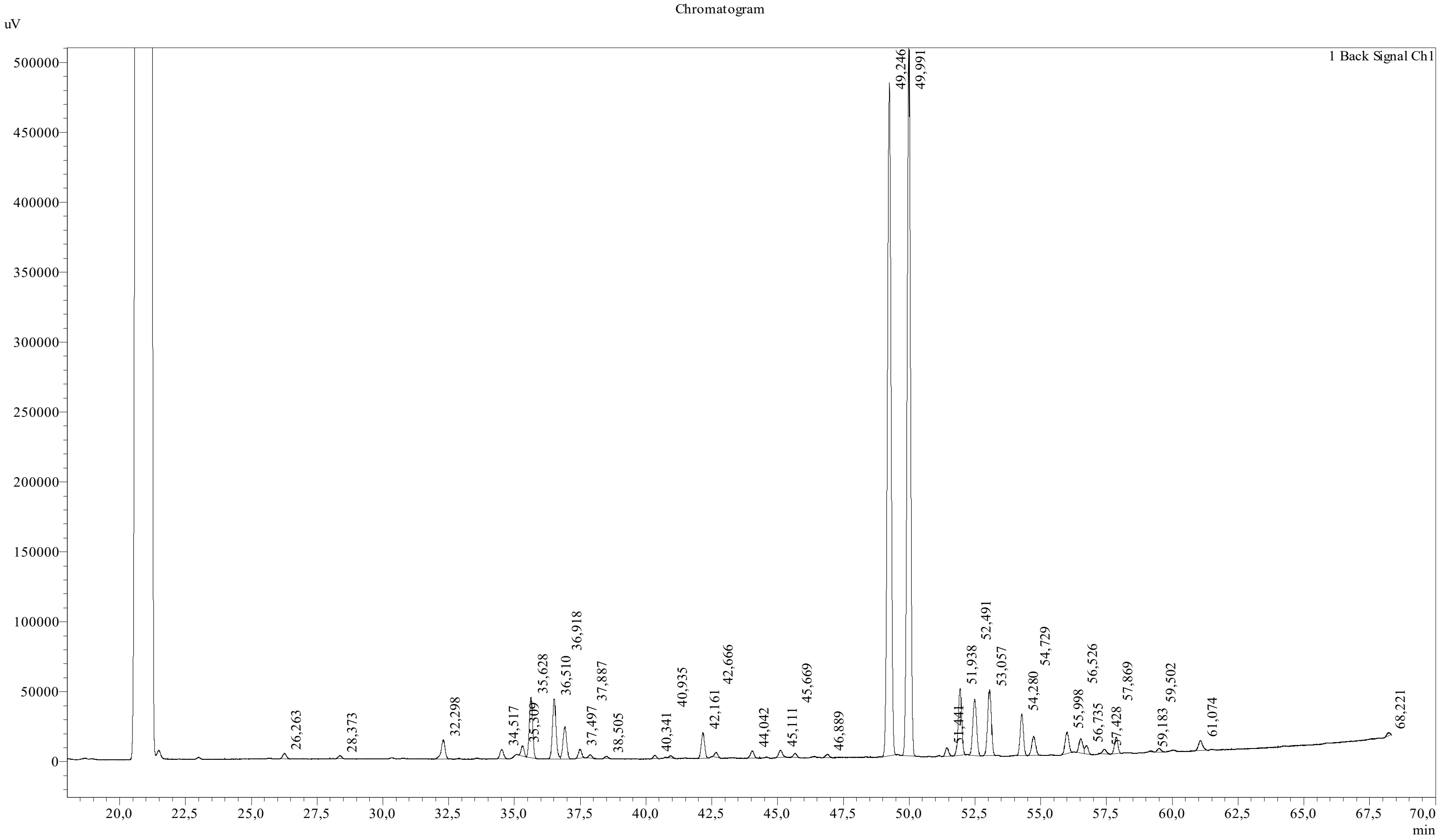

1.2.3.2. Lavender 2

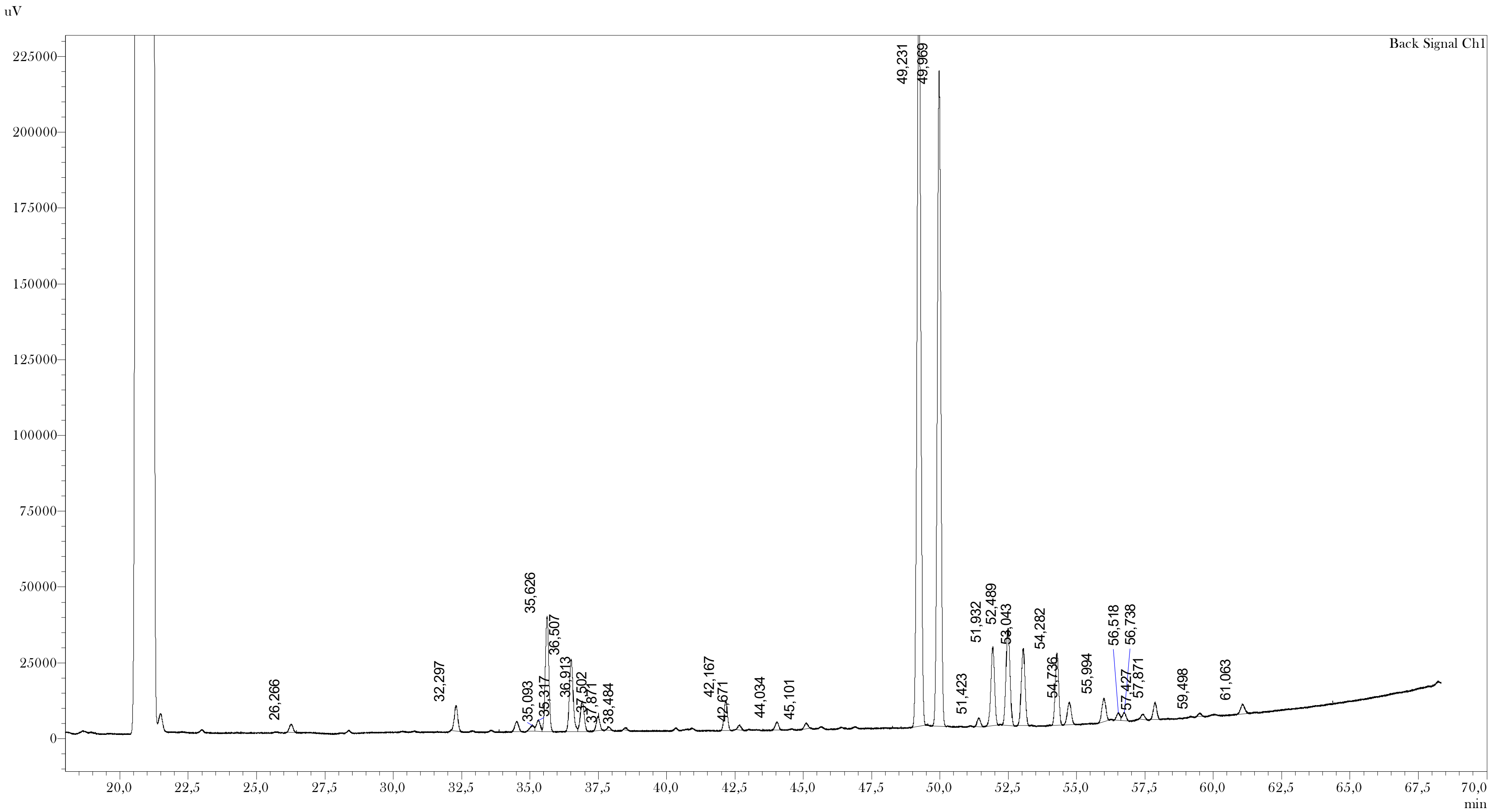
uV
Back Signal Ch1
225000
200000
175000
150000
125000
100000
75000
50000
25000
0
26,266
32,297
35,093
35,317
35,626
36,507
36,913
37,502
37,871
38,484
42,167
42,671
44,034
45,101
49,231
49,969
51,423
51,932
52,489
53,043
54,282
54,736
55,994
56,518
56,738
57,427
57,871
59,498
61,063
20,0
22,5
25,0
27,5
30,0
32,5
35,0
37,5
40,0
42,5
45,0
47,5
50,0
52,5
55,0
57,5
60,0
62,5
65,0
67,5
70,0
min

2. Additional tested lavender

Two pesticide-free lavender EOs were also included in the study. Lavender 3 comes from France (Drôme) as lavender 1 and lavender 4 comes from Bulgaria as lavender 2 (Table S1).

Table S1. Information on tested lavender EOs.

	Lavender 1	Lavender 2	Lavender 3	Lavender 4
Origins	France	Bulgaria	France	Bulgaria
Pesticides	Chlorpyrifos	ND	Chlorpyrifos	ND

Lavender 3 and 4 have been analyzed in the same conditions than lavender EOs 1 and 2.
Lavender 3 had non cytotoxic effect at the tested concentrations (Figure S1), whereas lavender 4 triggered a 40% loss of cell viability (Figure S1).

Lavender 3 had no effect on progesterone, estradiol, h-hCG and hPL secretion (Figure S2-S5). Lavender 4 had no effect on progesterone, h-hCG and hPL release but induced a significant reduction of estradiol release (x0.53 at $0.17x10^{-3}$%, Figure S3).

Two lavender EOs had no effect on P2X7 receptor activation (Figure S6).

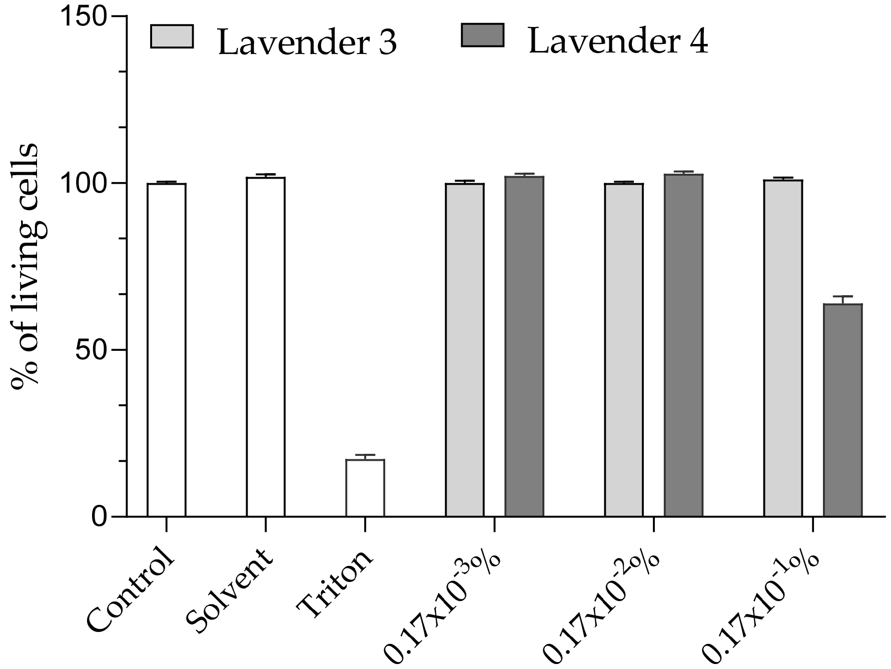

Figure S1. Cell viability was evaluated using the alamar blue assay after incubation of JEG-Tox cells with lavender EOs (3 and 4) at $0.17x10^{-3}$%, $0.17x10^{-2}$% and $0.17x10^{-1}$% for 72 h. Triton® X-100 at 0.016%, is used as a positive control for cytotoxicity.

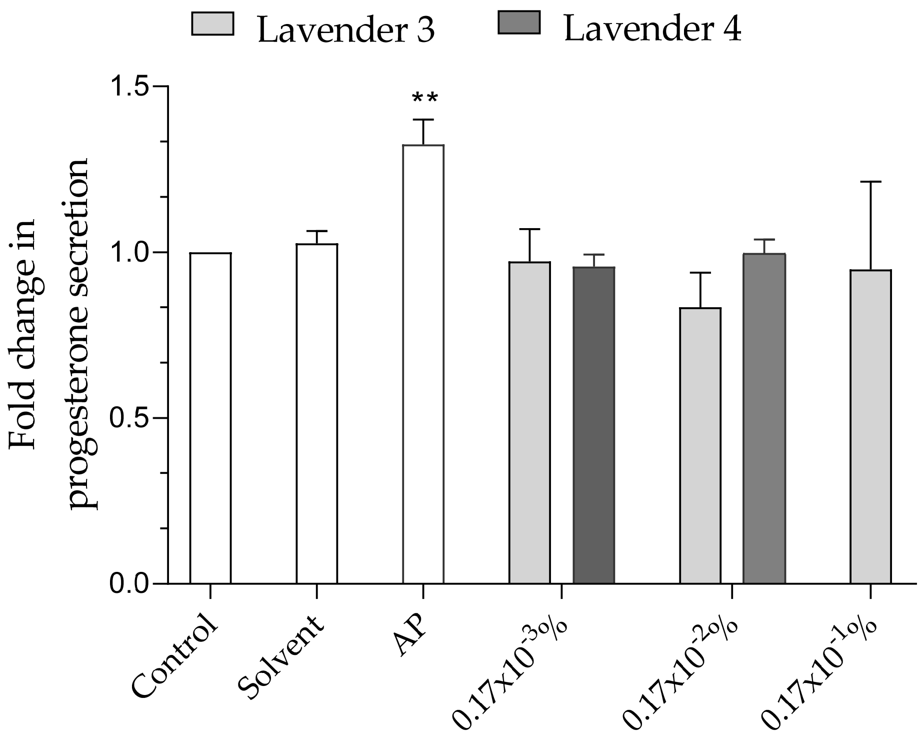

Figure S2. Quantification of progesterone release after incubation of JEG-Tox cells with lavender EOs (3 and 4) at 0.17x10^{-3}%, 0.17x10^{-2}% and 0.17x10^{-1}% for 72 h. 4-tert-amylphenol (AP) at 10μM is used as a positive control for progesterone release.

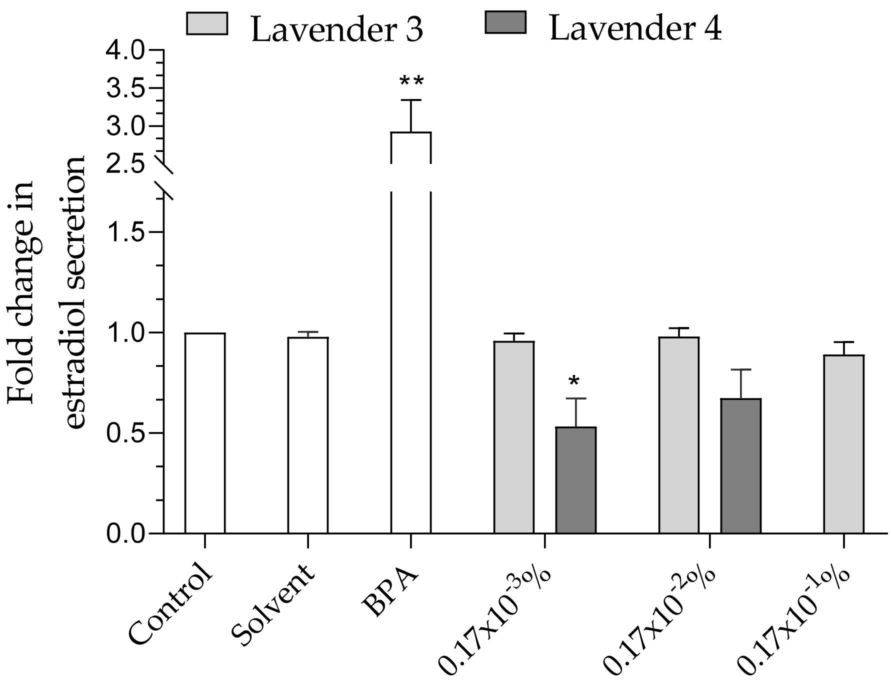

Figure S3. Quantification of estradiol release after incubation of JEG-Tox cells with lavender EOs (3 and 4) at 0.17x10^{-3}%, 0.17x10^{-2}% and 0.17x10^{-1}% for 72 h. Bisphenol A (BPA) at 20μM is used as a positive control for estradiol release.

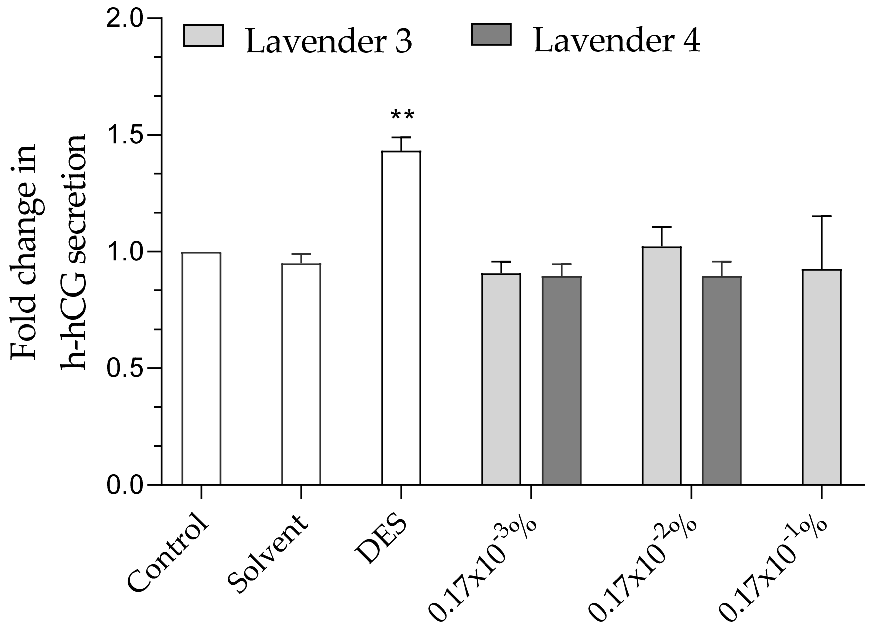

Figure S4. Quantification of h-hCG release after incubation of JEG-Tox cells with lavender EOs (3 and 4) at $0.17x10^{-3}\%$, $0.17x10^{-2}\%$ and $0.17x10^{-1}\%$ for 72 h. Diethylstilbestrol (DES) at 3.75μM is used as a positive control for h-hCG release.

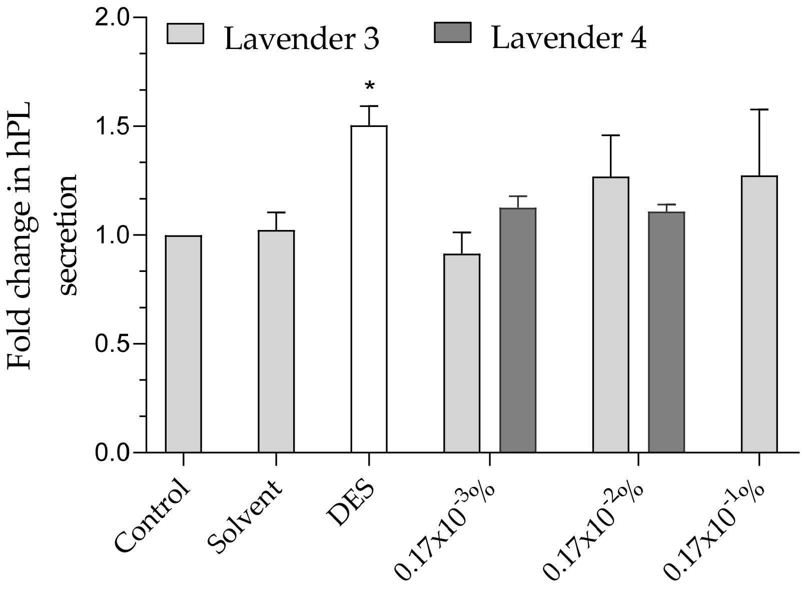

Figure S5. Quantification of hPL release after incubation of JEG-Tox cells with lavender EOs (3 and 4) at $0.17x10^{-3}\%$, $0.17x10^{-2}\%$ and $0.17x10^{-1}\%$ for 72 h. Diethylstilbestrol (DES) at 3.75μM is used as a positive control for hPL release.

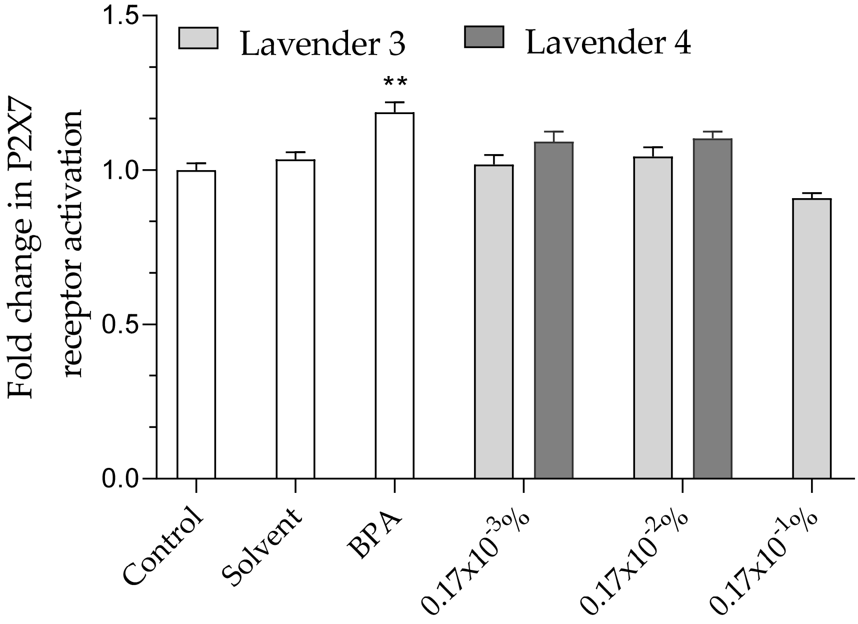

Figure S6. P2X7 receptor activation was evaluated using the YO-PRO-1 assay after incubation of JEG-Tox cells with lavender EO (3 and 4) at 0.17x10-3%, 0.17x10-2% and 0.17x10-1% for 72 h. Bisphenol A (BPA) at 20µM is used as a positive control for P2X7 receptor activation.
