# Supplementary material for: Cocktail Effect of Endocrine Disrupting Chemicals: Application to Chlorpyrifos in Lavender Essential Oils
Source: Int J Environ Res Public Health. 2022 Oct 10;19(19):12984. doi: 10.3390/ijerph191912984 (PMC9566273; doi:10.3390/ijerph191912984)
Supplement: Supplementary file 1 [file ijerph-19-12984-s001.zip › supplemental data 1.pdf]

Phytocontrol
AGRIFOOD

LAVENDER ESSENTIAL OIL 1

Pesticides

Multirésidus GC 250
FB3/02.c vers. 24 (01/02/2019)

Unité ◊ : mg/kg

	Résultat	LQ	méthode
1,4-Dimethylnaphtalene	ND	0,01	MOC3/05
2,4,6 trichlorophenol (TCP) (	ND	0,01	MOC3/05
2-Phenylphenol(somme)	ND		
2-Methoxybiphenyl	ND	0,01	MOC3/05
2-Phenylhydroquinone	ND	0,01	MOC3/05
2-Phenylphenol	ND	0,01	MOC3/05
3,4-dichloroaniline	ND	0,01	MOC3/05
4,4-Dichlorobenzophenone	ND	0,01	MOC3/05
Acetochlore	ND	0,01	MOC3/05
Acibenzolar-S-methyl (m)	ND	0,01	MOC3/05
Aclonifen	ND	0,01	MOC3/05
Acrinathrine	ND	0,01	MOC3/05
Alachlore	ND	0,01	MOC3/05
Ametryn	ND	0,01	MOC3/05
Amisulbrom	ND	0,01	MOC3/05
Atrazine	ND	0,01	MOC3/05
Benalaxyl dont Benalaxyl-M	ND	0,01	MOC3/05
Bendiocarb	ND	0,01	MOC3/05
Benfluraline	ND	0,01	MOC3/05
Benoxacor	ND	0,01	MOC3/05
Bifenox	ND	0,01	MOC3/05
Bifenthrine (∑ des isomères)	ND	0,01	MOC3/05
Biphenyl	ND	0,01	MOC3/05
Bitertanol (Σ des isomères)	ND	0,01	MOC3/05
Bromocyclen	ND	0,01	MOC3/05
Bromophos-ethyl	ND	0,01	MOC3/05
Bromophos-methyl	ND	0,01	MOC3/05
Bromopropylate	ND	0,01	MOC3/05
Butachlor	ND	0,01	MOC3/05
Butraline	ND	0,01	MOC3/05
Captafol	ND	0,01	MOC3/05
Captan(somme)	ND		
Captan	ND	0,01	MOC3/05
Tetrahydrophtalimide (THF	ND	0,01	MOC3/05
Carbaryl	ND	0,01	MOC3/05
Carbofuran(somme GC) (m)	ND		
Carbofuran	ND	0,01	MOC3/05
Carbofuran-3-Hydroxy	ND	0,01	MOC3/05
Furathiocarbe	ND	0,01	MOC3/05
Carbophenothion	ND	0,01	MOC3/05
Carfentrazone-ethyl	ND	0,01	MOC3/05
Chlorbenside	ND	0,01	MOC3/05
Chlordane(cis+trans)	ND	0,01	MOC3/05
Chlorfenapyr	ND	0,01	MOC3/05
Chlorfenson	ND	0,01	MOC3/05
Chlorfenvinphos	ND	0,01	MOC3/05
Chlorobenzilate	ND	0,01	MOC3/05
Chlorothalonil	ND	0,01	MOC3/05
Chlorprophame	ND	0,01	MOC3/05
Chlorpyrifos	0,014	0,01	MOC3/05
Chlorpyrifos-methyl	ND	0,01	MOC3/05
Chlorthal dimethyl	ND	0,01	MOC3/05
Chlorthiophos	ND	0,01	MOC3/05
Chlozolinate	ND	0,01	MOC3/05
Clomazone	ND	0,01	MOC3/05
Coumaphos	ND	0,01	MOC3/05
Cyfluthrine (β+γ)	ND	0,01	MOC3/05
Cyhalofop-butyl	ND	0,01	MOC3/05
Cyhalothrine(∑ des isomères	ND	0,01	MOC3/05
Cymiazole	ND	0,01	MOC3/05
Cypermethrine(α+β+θ+ζ)	ND	0,01	MOC3/05
Cyproconazole	ND	0,01	MOC3/05
Cyprodinil	ND	0,01	MOC3/05
DDT(somme)	ND		
o,p'-DDT	ND	0,01	MOC3/05
p,p'-DDT	ND	0,01	MOC3/05
p,p'-DDE	ND	0,01	MOC3/05
p,p'-TDE(DDD)	ND	0,01	MOC3/05
Deltamethrine	ND	0,01	MOC3/05
Demeton-S-methyl	ND	0,01	MOC3/05
Dialifos	ND	0,01	MOC3/05
Dichlobenil	ND	0,01	MOC3/05
Dichlofenthion	ND	0,01	MOC3/05
Dichlofluanide	ND	0,01	MOC3/05
Dichlorvos	ND	0,01	MOC3/05
Diclofop-methyl (m)	ND	0,01	MOC3/05
Dicofol(Σ des isomères)	ND		
Dicofol o,p'	ND	0,01	MOC3/05
Dicofol p,p'	ND	0,01	MOC3/05
Dicrotophos	ND	0,01	MOC3/05
Dieldrin(somme)	ND		
Aldrin	ND	0,01	MOC3/05
Dieldrin	ND	0,01	MOC3/05
Diethofencarb	ND	0,01	MOC3/05
Difenoconazole	ND	0,01	MOC3/05
Diflufenican	ND	0,01	MOC3/05
Dimetachlor	ND	0,01	MOC3/05
Dinitramine	ND	0,01	MOC3/05
Diphenylamine	ND	0,01	MOC3/05
Disulfoton (m)	ND	0,01	MOC3/05
Ditalimfos	ND	0,01	MOC3/05
Edifenphos	ND	0,01	MOC3/05
Endosulfan(somme)	ND		
Endosulfan α	ND	0,01	MOC3/05
Endosulfan β	ND	0,01	MOC3/05
Endosulfan sulfate	ND	0,01	MOC3/05
Endrin	ND	0,01	MOC3/05
Endrin-ketone	ND	0,01	MOC3/05
EPN	ND	0,01	MOC3/05
Ethalfluraline	ND	0,01	MOC3/05
Ethiofencarb	ND	0,01	MOC3/05
Ethion	ND	0,01	MOC3/05
Ethofumesate (m)	ND	0,01	MOC3/05
Ethoprophos	ND	0,01	MOC3/05
Ethoxyquine	ND	0,01	MOC3/05
Etofenprox	ND	0,01	MOC3/05
Etridiazole	ND	0,01	MOC3/05
Etrimfos	ND	0,01	MOC3/05
Famoxadone	ND	0,01	MOC3/05
Famphur	ND	0,01	MOC3/05
Fenamiphos (m)	ND	0,01	MOC3/05
Fenarimol	ND	0,01	MOC3/05
Fenazaquin	ND	0,01	MOC3/05
Fenchlorphos (m)	ND	0,01	MOC3/05
Fenhexamide	ND	0,01	MOC3/05
Fenitrothion	ND	0,01	MOC3/05
Fenobucarbe	ND	0,01	MOC3/05
Fenpropathrine	ND	0,01	MOC3/05
Fenpropimorphe (∑ des isomères)	ND	0,01	MOC3/05
Fenvalerate (Σ des isomères)	ND	0,01	MOC3/05
Fipronil(somme)	ND		
Fipronil	ND	0,005	MOC3/05
Fipronil-sulfone	ND	0,005	MOC3/05
Fipronil-desulfinyl	ND	0,01	MOC3/05
Fluazifop-p-butyl (m)	ND	0,01	MOC3/05
Fluchloralin	ND	0,01	MOC3/05
Flucythrinate	ND	0,01	MOC3/05
Fludioxonil	ND	0,01	MOC3/05
Flufenacet (m)	ND	0,01	MOC3/05
Fluopicolide	ND	0,01	MOC3/05
Flurochloridone	ND	0,01	MOC3/05
Fluroxypyr-methylheptyl ester (m)	ND	0,01	MOC3/05
Flusilazole	ND	0,01	MOC3/05
Flutolanil	ND	0,01	MOC3/05
Flutriafol	ND	0,01	MOC3/05
Fluvalinate (Tau)	ND	0,01	MOC3/05
Folpet(somme)	ND		
Folpet	ND	0,01	MOC3/05
Phtalimide	ND	0,01	MOC3/05
Fonofos	ND	0,01	MOC3/05
Formothion	ND	0,01	MOC3/05
Furalaxyl	ND	0,01	MOC3/05
Haloxyfop-2-ethoxyethyl (m)	ND	0,01	MOC3/05
Haloxyfop-methyl(R+S) (m)	ND	0,01	MOC3/05
HCB	ND	0,01	MOC3/05
HCH alpha	ND	0,01	MOC3/05
HCH beta	ND	0,01	MOC3/05
HCH gamma	ND	0,01	MOC3/05
Heptachlore(somme)	ND		
Heptachlore	ND	0,01	MOC3/05
Heptachlore epoxyde cis-	ND	0,01	MOC3/05
Heptachlore epoxyde trans	ND	0,01	MOC3/05
Heptenophos	ND	0,01	MOC3/05
Hexazinone	ND	0,01	MOC3/05
Iodofenphos	ND	0,01	MOC3/05
Iprodione	ND	0,01	MOC3/05
Isobenzan	ND	0,01	MOC3/05
Isodrine	ND	0,01	MOC3/05
Isofenphos-ethyl	ND	0,01	MOC3/05
Isofenphos-methyl	ND	0,01	MOC3/05
Isoxadifen-ethyl	ND	0,01	MOC3/05
Leptophos	ND	0,01	MOC3/05
Malathion(somme)	ND		
Malathion	ND	0,01	MOC3/05
Malaoxon	ND	0,01	MOC3/05
Mepanipyrim	ND	0,01	MOC3/05
Mepronil	ND	0,01	MOC3/05
Metalaxyl dont Metalaxyl-M	ND	0,01	MOC3/05
Metazachlor	ND	0,01	MOC3/05
Methacrifos	ND	0,01	MOC3/05
Methidathion	ND	0,01	MOC3/05
Methoxychlore	ND	0,01	MOC3/05
Metolachlore dont S-Metolachlore	ND	0,01	MOC3/05
Mirex	ND	0,01	MOC3/05
Myclobutanil	ND	0,01	MOC3/05

Nitrofene	ND	0,01	MOC3/05	Tebufenpyrad	ND	0,01	MOC3/05
Nitrothal isopropyle	ND	0,01	MOC3/05	Tebupirimphos	ND	0,01	MOC3/05
Oxadiazon	ND	0,01	MOC3/05	Tecnazene	ND	0,01	MOC3/05
Oxadixyl	ND	0,01	MOC3/05	Tefluthrine	ND	0,01	MOC3/05
Oxyfluorfene	ND	0,01	MOC3/05	Terbacil	ND	0,01	MOC3/05
Parathion-ethyl	ND	0,01	MOC3/05	Terbufos	ND	0,01	MOC3/05
Parathion-methyl (m)	ND	0,01	MOC3/05	Terbuthylazine	ND	0,01	MOC3/05
PCB 028	ND	0,01	MOC3/05	Terbutryne	ND	0,01	MOC3/05
PCB 052	ND	0,01	MOC3/05	Tetrachlorvinphos	ND	0,01	MOC3/05
PCB 101	ND	0,01	MOC3/05	Tetradifon	ND	0,01	MOC3/05
PCB 118	ND	0,01	MOC3/05	Tetramethrine	ND	0,01	MOC3/05
PCB 138	ND	0,01	MOC3/05	Tetrasul	ND	0,01	MOC3/05
PCB 153	ND	0,01	MOC3/05	Tolclofos-methyl	ND	0,01	MOC3/05
PCB 180	ND	0,01	MOC3/05	Tolylfluanid (m)	ND	0,01	MOC3/05
Penconazole	ND	0,01	MOC3/05	Tralomethrine	ND	0,01	MOC3/05
Pendimethaline	ND	0,01	MOC3/05	Transfluthrine	ND	0,01	MOC3/05
Pentachloroanisole	ND	0,01	MOC3/05	Triadimefon	ND	0,01	MOC3/05
Permethrine(cis + trans)	ND	0,01	MOC3/05	Triadimenol	ND	0,01	MOC3/05
Perthane	ND	0,01	MOC3/05	Triallate	ND	0,01	MOC3/05
Phenothrine	ND	0,01	MOC3/05	Triamiphos	ND	0,01	MOC3/05
Phenthoate	ND	0,01	MOC3/05	Triazophos	ND	0,01	MOC3/05
Phosalone	ND	0,01	MOC3/05	Trichloronat	ND	0,01	MOC3/05
Piperonyl butoxide	ND	0,01	MOC3/05	Trifluraline	ND	0,01	MOC3/05
Pirimicarb	ND	0,01	MOC3/05	Valifenalate	ND	0,01	MOC3/05
Pirimiphos-ethyl	ND	0,01	MOC3/05	Vinclozoline	ND	0,01	MOC3/05
Pirimiphos-methyl	ND	0,01	MOC3/05	Zoxamide	ND	0,01	MOC3/05
Plifenate	ND	0,01	MOC3/05				
Pretilachlore	ND	0,01	MOC3/05				
Procymidone	ND	0,01	MOC3/05				
Profenophos	ND	0,01	MOC3/05				
Prometryn	ND	0,01	MOC3/05				
Propachlore (m)	ND	0,01	MOC3/05				
Propazine	ND	0,01	MOC3/05				
Propetamphos	ND	0,01	MOC3/05				
Prophame	ND	0,01	MOC3/05				
Propiconazole	ND	0,01	MOC3/05				
Propyzamide	ND	0,01	MOC3/05				
Proquinazid	ND	0,01	MOC3/05				
Prosulfocarbe	ND	0,01	MOC3/05				
Prothiophos	ND	0,01	MOC3/05				
Prothoate	ND	0,01	MOC3/05				
Pyrazophos	ND	0,01	MOC3/05				
Pyridaben	ND	0,01	MOC3/05				
Pyridalyl	ND	0,01	MOC3/05				
Pyridaphenthion	ND	0,01	MOC3/05				
Pyrifenox	ND	0,01	MOC3/05				
Pyrimethanil	ND	0,01	MOC3/05				
Pyriproxyfen	ND	0,01	MOC3/05				
Quinalphos	ND	0,01	MOC3/05				
Quinomethionate	ND	0,01	MOC3/05				
Quinoxyfen	ND	0,01	MOC3/05				
Quintozene(somme)	ND						
Quintozene	ND	0,01	MOC3/05				
Pentachloroaniline (PCA)	ND	0,01	MOC3/05				
Quizalofop-ethyl	ND	0,01	MOC3/05				
S 421	ND	0,01	MOC3/05				
Sebuthylazine	ND	0,01	MOC3/05				
Secbumeton	ND	0,01	MOC3/05				
Sulfotep	ND	0,01	MOC3/05				
Sulprofos	ND	0,01	MOC3/05				
Tebuconazole	ND	0,01	MOC3/05				

Pesticides

Multirésidus GC 250

FB3/02.c vers. 24 (01/02/2019)

**Unité ⁰ : mg/kg**	Résultat	LQ	méthode
1,4-Dimethylnaphtalene	ND	0,01	MOC3/05
2,4,6 trichlorophenol (TCP) (ı	ND	0,01	MOC3/05
2-Phenylphenol(somme)	ND		
2-Methoxybiphenyl	ND	0,01	MOC3/05
2-Phenylhydroquinone	ND	0,01	MOC3/05
2-Phenylphenol	ND	0,01	MOC3/05
3,4-dichloroaniline	ND	0,01	MOC3/05
4,4-Dichlorobenzophenone	ND	0,01	MOC3/05
Acetochlore	ND	0,01	MOC3/05
Acibenzolar-S-methyl (m)	ND	0,01	MOC3/05
Aclonifen	ND	0,01	MOC3/05
Acrinathrine	ND	0,01	MOC3/05
Alachlore	ND	0,01	MOC3/05
Ametryn	ND	0,01	MOC3/05
Amisulbrom	ND	0,01	MOC3/05
Atrazine	ND	0,01	MOC3/05
Benalaxyl dont Benalaxyl-M	ND	0,01	MOC3/05
Bendiocarb	ND	0,01	MOC3/05
Benfluraline	ND	0,01	MOC3/05
Benoxacor	ND	0,01	MOC3/05
Bifenox	ND	0,01	MOC3/05
Bifenthrine (∑ des isomères)	ND	0,01	MOC3/05
Biphenyl	ND	0,01	MOC3/05
Bitertanol (Σ des isomères)	ND	0,01	MOC3/05
Bromocyclen	ND	0,01	MOC3/05
Bromophos-ethyl	ND	0,01	MOC3/05
Bromophos-methyl	ND	0,01	MOC3/05
Bromopropylate	ND	0,01	MOC3/05
Butachlor	ND	0,01	MOC3/05
Butraline	ND	0,01	MOC3/05
Captafol	ND	0,01	MOC3/05
Captan(somme)	ND		
Captan	ND	0,01	MOC3/05
Tetrahydrophtalimide (THF	ND	0,01	MOC3/05
Carbaryl	ND	0,01	MOC3/05
Carbofuran(somme GC) (m)	ND		
Carbofuran	ND	0,01	MOC3/05
Carbofuran-3-Hydroxy	ND	0,01	MOC3/05
Furathiocarbe	ND	0,01	MOC3/05
Carbophenothion	ND	0,01	MOC3/05
Carfentrazone-ethyl	ND	0,01	MOC3/05
Chlorbenside	ND	0,01	MOC3/05
Chlordane(cis+trans)	ND	0,01	MOC3/05
Chlorfenapyr	ND	0,01	MOC3/05
Chlorfenson	ND	0,01	MOC3/05
Chlorfenvinphos	ND	0,01	MOC3/05
Chlorobenzilate	ND	0,01	MOC3/05
Chlorothalonil	ND	0,01	MOC3/05
Chlorprophame	ND	0,01	MOC3/05
Chlorpyrifos	0,014	0,01	MOC3/05
Chlorpyrifos-methyl	ND	0,01	MOC3/05
Chlorthal dimethyl	ND	0,01	MOC3/05
Chlorthiophos	ND	0,01	MOC3/05
Chlozolinate	ND	0,01	MOC3/05
Clomazone	ND	0,01	MOC3/05

Coumaphos	ND	0,01	MOC3/05
Cyfluthrine (β+γ)	ND	0,01	MOC3/05
Cyhalofop-butyl	ND	0,01	MOC3/05
Cyhalothrine(∑ des isomères	ND	0,01	MOC3/05
Cymiazole	ND	0,01	MOC3/05
Cypermethrine(α+β+θ+ζ)	ND	0,01	MOC3/05
Cyproconazole	ND	0,01	MOC3/05
Cyprodinil	ND	0,01	MOC3/05
DDT(somme)	ND		
o,p'-DDT	ND	0,01	MOC3/05
p,p'-DDT	ND	0,01	MOC3/05
p,p'-DDE	ND	0,01	MOC3/05
p,p'-TDE(DDD)	ND	0,01	MOC3/05
Deltamethrine	ND	0,01	MOC3/05
Demeton-S-methyl	ND	0,01	MOC3/05
Dialifos	ND	0,01	MOC3/05
Dichlobenil	ND	0,01	MOC3/05
Dichlofenthion	ND	0,01	MOC3/05
Dichlofluanide	ND	0,01	MOC3/05
Dichlorvos	ND	0,01	MOC3/05
Diclofop-methyl (m)	ND	0,01	MOC3/05
Dicofol(Σ des isomères)	ND		
Dicofol o,p'	ND	0,01	MOC3/05
Dicofol p,p'	ND	0,01	MOC3/05
Dicrotophos	ND	0,01	MOC3/05
Dieldrin(somme)	ND		
Aldrin	ND	0,01	MOC3/05
Dieldrin	ND	0,01	MOC3/05
Diethofencarb	ND	0,01	MOC3/05
Difenoconazole	ND	0,01	MOC3/05
Diflufenican	ND	0,01	MOC3/05
Dimetachlor	ND	0,01	MOC3/05
Dinitramine	ND	0,01	MOC3/05
Diphenylamine	ND	0,01	MOC3/05
Disulfoton (m)	ND	0,01	MOC3/05
Ditalimfos	ND	0,01	MOC3/05
Edifenphos	ND	0,01	MOC3/05
Endosulfan(somme)	ND		
Endosulfan α	ND	0,01	MOC3/05
Endosulfan β	ND	0,01	MOC3/05
Endosulfan sulfate	ND	0,01	MOC3/05
Endrin	ND	0,01	MOC3/05
Endrin-ketone	ND	0,01	MOC3/05
EPN	ND	0,01	MOC3/05
Ethalfluraline	ND	0,01	MOC3/05
Ethiofencarb	ND	0,01	MOC3/05
Ethion	ND	0,01	MOC3/05
Ethofumesate (m)	ND	0,01	MOC3/05
Ethoprophos	ND	0,01	MOC3/05
Ethoxyquine	ND	0,01	MOC3/05
Etofenprox	ND	0,01	MOC3/05
Etridiazole	ND	0,01	MOC3/05
Etrimfos	ND	0,01	MOC3/05
Famoxadone	ND	0,01	MOC3/05
Famphur	ND	0,01	MOC3/05
Fenamiphos (m)	ND	0,01	MOC3/05
Fenarimol	ND	0,01	MOC3/05
Fenazaquin	ND	0,01	MOC3/05
Fenchlorphos (m)	ND	0,01	MOC3/05
Fenhexamide	ND	0,01	MOC3/05
Fenitrothion	ND	0,01	MOC3/05

Fenobucarbe	ND	0,01	MOC3/05
Fenpropathrine	ND	0,01	MOC3/05
Fenpropimorphe (∑ des isomères)	ND	0,01	MOC3/05
Fenvalerate (Σ des isomères;	ND	0,01	MOC3/05
Fipronil(somme)	ND		
Fipronil	ND	0,005	MOC3/05
Fipronil-sulfone	ND	0,005	MOC3/05
Fipronil-desulfinyl	ND	0,01	MOC3/05
Fluazifop-p-butyl (m)	ND	0,01	MOC3/05
Fluchloralin	ND	0,01	MOC3/05
Flucythrinate	ND	0,01	MOC3/05
Fludioxonil	ND	0,01	MOC3/05
Flufenacet (m)	ND	0,01	MOC3/05
Fluopicolide	ND	0,01	MOC3/05
Flurochloridone	ND	0,01	MOC3/05
Fluroxypyr-methylheptyl ester (m)	ND	0,01	MOC3/05
Flusilazole	ND	0,01	MOC3/05
Flutolanil	ND	0,01	MOC3/05
Flutriafol	ND	0,01	MOC3/05
Fluvalinate (Tau)	ND	0,01	MOC3/05
Folpet(somme)	ND		
Folpet	ND	0,01	MOC3/05
Phtalimide	ND	0,01	MOC3/05
Fonofos	ND	0,01	MOC3/05
Formothion	ND	0,01	MOC3/05
Furalaxyl	ND	0,01	MOC3/05
Haloxyfop-2-ethoxyethyl (m)	ND	0,01	MOC3/05
Haloxyfop-methyl(R+S) (m)	ND	0,01	MOC3/05
HCB	ND	0,01	MOC3/05
HCH alpha	ND	0,01	MOC3/05
HCH beta	ND	0,01	MOC3/05
HCH gamma	ND	0,01	MOC3/05
Heptachlore(somme)	ND		
Heptachlore	ND	0,01	MOC3/05
Heptachlore epoxyde cis-	ND	0,01	MOC3/05
Heptachlore epoxyde trans	ND	0,01	MOC3/05
Heptenophos	ND	0,01	MOC3/05
Hexazinone	ND	0,01	MOC3/05
Iodofenphos	ND	0,01	MOC3/05
Iprodione	ND	0,01	MOC3/05
Isobenzan	ND	0,01	MOC3/05
Isodrine	ND	0,01	MOC3/05
Isofenphos-ethyl	ND	0,01	MOC3/05
Isofenphos-methyl	ND	0,01	MOC3/05
Isoxadifen-ethyl	ND	0,01	MOC3/05
Leptophos	ND	0,01	MOC3/05
Malathion(somme)	ND		
Malathion	ND	0,01	MOC3/05
Malaoxon	ND	0,01	MOC3/05
Mepanipyrim	ND	0,01	MOC3/05
Mepronil	ND	0,01	MOC3/05
Metalaxyl dont Metalaxyl-M	ND	0,01	MOC3/05
Metazachlor	ND	0,01	MOC3/05
Methacrifos	ND	0,01	MOC3/05
Methidathion	ND	0,01	MOC3/05
Methoxychlore	ND	0,01	MOC3/05
Metolachlore dont S-Metolachlore	ND	0,01	MOC3/05
Mirex	ND	0,01	MOC3/05
Myclobutanil	ND	0,01	MOC3/05

Nitrofene	ND	0,01	MOC3/05
Nitrothal isopropyle	ND	0,01	MOC3/05
Oxadiazon	ND	0,01	MOC3/05
Oxadixyl	ND	0,01	MOC3/05
Oxyfluorfene	ND	0,01	MOC3/05
Parathion-ethyl	ND	0,01	MOC3/05
Parathion-methyl (m)	ND	0,01	MOC3/05
PCB 028	ND	0,01	MOC3/05
PCB 052	ND	0,01	MOC3/05
PCB 101	ND	0,01	MOC3/05
PCB 118	ND	0,01	MOC3/05
PCB 138	ND	0,01	MOC3/05
PCB 153	ND	0,01	MOC3/05
PCB 180	ND	0,01	MOC3/05
Penconazole	ND	0,01	MOC3/05
Pendimethaline	ND	0,01	MOC3/05
Pentachloroanisole	ND	0,01	MOC3/05
Permethrine(cis + trans)	ND	0,01	MOC3/05
Perthane	ND	0,01	MOC3/05
Phenothrine	ND	0,01	MOC3/05
Phenthoate	ND	0,01	MOC3/05
Phosalone	ND	0,01	MOC3/05
Piperonyl butoxide	ND	0,01	MOC3/05
Pirimicarb	ND	0,01	MOC3/05
Pirimiphos-ethyl	ND	0,01	MOC3/05
Pirimiphos-methyl	ND	0,01	MOC3/05
Plifenate	ND	0,01	MOC3/05
Pretilachlore	ND	0,01	MOC3/05
Procymidone	ND	0,01	MOC3/05
Profenophos	ND	0,01	MOC3/05
Prometryn	ND	0,01	MOC3/05
Propachlore (m)	ND	0,01	MOC3/05
Propazine	ND	0,01	MOC3/05
Propetamphos	ND	0,01	MOC3/05
Prophame	ND	0,01	MOC3/05
Propiconazole	ND	0,01	MOC3/05
Propyzamide	ND	0,01	MOC3/05
Proquinazid	ND	0,01	MOC3/05
Prosulfocarbe	ND	0,01	MOC3/05
Prothiophos	ND	0,01	MOC3/05
Prothoate	ND	0,01	MOC3/05
Pyrazophos	ND	0,01	MOC3/05
Pyridaben	ND	0,01	MOC3/05
Pyridalyl	ND	0,01	MOC3/05
Pyridaphenthion	ND	0,01	MOC3/05
Pyrifenox	ND	0,01	MOC3/05
Pyrimethanil	ND	0,01	MOC3/05
Pyriproxyfen	ND	0,01	MOC3/05
Quinalphos	ND	0,01	MOC3/05
Quinomethionate	ND	0,01	MOC3/05
Quinoxyfen	ND	0,01	MOC3/05
Quintozene(somme)	ND		
Quintozene	ND	0,01	MOC3/05
Pentachloroaniline (PCA)	ND	0,01	MOC3/05
Quizalofop-ethyl	ND	0,01	MOC3/05
S 421	ND	0,01	MOC3/05
Sebuthylazine	ND	0,01	MOC3/05
Secbumeton	ND	0,01	MOC3/05
Sulfotep	ND	0,01	MOC3/05
Sulprofos	ND	0,01	MOC3/05
Tebuconazole	ND	0,01	MOC3/05

Tebufenpyrad	ND	0,01	MOC3/05
Tebupirimphos	ND	0,01	MOC3/05
Tecnazene	ND	0,01	MOC3/05
Tefluthrine	ND	0,01	MOC3/05
Terbacil	ND	0,01	MOC3/05
Terbufos	ND	0,01	MOC3/05
Terbuthylazine	ND	0,01	MOC3/05
Terbutryne	ND	0,01	MOC3/05
Tetrachlorvinphos	ND	0,01	MOC3/05
Tetradifon	ND	0,01	MOC3/05
Tetramethrine	ND	0,01	MOC3/05
Tetrasul	ND	0,01	MOC3/05
Tolclofos-methyl	ND	0,01	MOC3/05
Tolylfluanid (m)	ND	0,01	MOC3/05
Tralomethrine	ND	0,01	MOC3/05
Transfluthrine	ND	0,01	MOC3/05
Triadimefon	ND	0,01	MOC3/05
Triadimenol	ND	0,01	MOC3/05
Triallate	ND	0,01	MOC3/05
Triamiphos	ND	0,01	MOC3/05
Triazophos	ND	0,01	MOC3/05
Trichloronat	ND	0,01	MOC3/05
Trifluraline	ND	0,01	MOC3/05
Valifenalate	ND	0,01	MOC3/05
Vinclozoline	ND	0,01	MOC3/05
Zoxamide	ND	0,01	MOC3/05
